# Image quality of DWI at breast MRI depends on the amount of fibroglandular tissue: implications for unenhanced screening

**DOI:** 10.1007/s00330-023-10321-y

**Published:** 2023-11-27

**Authors:** Mirjam Wielema, Paul E. Sijens, Ruud M. Pijnappel, Geertruida H. De Bock, Marcel Zorgdrager, Marius G. J. Kok, Eva Rainer, Raoul Varga, Paola Clauser, Matthijs Oudkerk, Monique D. Dorrius, Pascal A. T. Baltzer

**Affiliations:** 1 Department of Radiology, University of Groningen, University Medical Center Groningen, Groningen, the Netherlands; 2 Department of Epidemiology, University of Groningen, University Medical Center Groningen, Groningen, the Netherlands; 3 Department of Radiology, Utrecht University, University Medical Center Utrecht, Utrecht, the Netherlands; 4 Department of Radiology, Medisch Spectrum Twente, Enschede, the Netherlands; 5 Department of Biomedical Imaging and Image-Guided Therapy, Medical University of Vienna, Vienna, Austria; 6 Institute for Diagnostic Accuracy, Groningen, the Netherlands

**Keywords:** Breast density, Diffusion magnetic resonance imaging, Area under the curve, Neoplasms, Early detection of cancer

## Abstract

**Objectives:**

To compare image quality of diffusion-weighted imaging (DWI) and contrast-enhanced breast MRI (DCE-T1) stratified by the amount of fibroglandular tissue (FGT) as a measure of breast density.

**Methods:**

Retrospective, multi-reader, bicentric visual grading analysis study on breast density (A–D) and overall image and fat suppression quality of DWI and DCE-T1, scored on a standard 5-point Likert scale. Cross tabulations and visual grading characteristic (VGC) curves were calculated for fatty breasts (A/B) versus dense breasts (C/D).

**Results:**

Image quality of DWI was higher in the case of increased breast density, with good scores (score 3–5) in 85.9% (D) and 88.4% (C), compared to 61.6% (B) and 53.5% (A). Overall image quality of DWI was in favor of dense breasts (C/D), with an area under the VGC curve of 0.659 (*p* < 0.001). Quality of DWI and DCE-T1 fat suppression increased with higher breast density, with good scores (score 3–5) for 86.9% and 45.7% of density D, and 90.2% and 42.9% of density C cases, compared to 76.0% and 33.6% for density B and 54.7% and 29.6% for density A (DWI and DCE-T1 respectively).

**Conclusions:**

Dense breasts show excellent fat suppression and substantially higher image quality in DWI images compared with non-dense breasts. These results support the setup of studies exploring DWI-based MR imaging without IV contrast for additional screening of women with dense breasts.

**Clinical relevance statement:**

Our findings demonstrate that image quality of DWI is robust in women with an increased amount of fibroglandular tissue, technically supporting the feasibility of exploring applications such as screening of women with mammographically dense breasts.

**Key Points:**

• *Image and fat suppression quality of diffusion-weighted imaging are dependent on the amount of fibroglandular tissue (FGT) which is closely connected to breast density.*

• *Fat suppression quality in diffusion-weighted imaging of the breast is best in women with a high amount of fibroglandular tissue.*

• *High image quality of diffusion-weighted imaging in women with a high amount of FGT in MRI supports that the technical feasibility of DWI can be explored in the additional screening of women with mammographically dense breasts.*

**Graphical Abstract:**

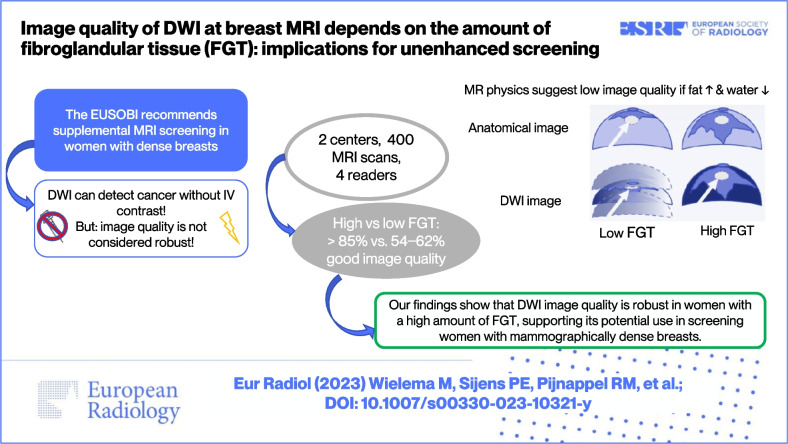

**Supplementary Information:**

The online version contains supplementary material available at 10.1007/s00330-023-10321-y.

## Introduction

Women with dense breasts (breast density C and D) constitute about 40% of the female screening population and pose an organizational challenge to any supplemental imaging program. Using supplemental imaging to screen for breast cancer is an active research topic and contrast-enhanced breast MRI is regarded as the most promising modality to compensate for the limited ability of mammography to detect breast cancer in women with extremely dense breast tissue (density D) [1].

Consequently, based on the results of the DENSE trial and the higher breast cancer risk in dense breasts, the EUSOBI has published recommendations in favor of breast MRI screening in women with extremely dense breasts [2, 3]. Contrast-enhanced breast MRI, however, is expensive and its application for screening is limited by the need to inject intravenous (IV) contrast and concerns regarding the perceived risk of adverse effects of gadolinium-based contrast media.

In addition, though not yet proven in a prospective trial, it is conceivable that MRI would improve screening also in women with moderately dense breasts (breast density C). Contrast-enhanced mammography as a competitor for breast MRI also requires IV contrast and increases exposure to ionizing radiation. In addition, the risk profile of iodine-based contrast media is less favorable than that of gadolinium-based contrast media. Contrast agent is not needed for diffusion-weighted imaging (DWI), an alternative MRI technique that visualizes and quantifies the Brownian molecular motion in the extracellular space in vivo. Breast cancer typically shows areas of hindered diffusion that appear hyperintense on diffusion-weighted images and can thus be used for cancer detection. The apparent diffusion coefficient is a DWI metric that can quantitatively distinguish benign from malignant findings. Initial research on DWI, which is acquired faster than a contrast-enhanced breast MRI protocol, has demonstrated favorable diagnostic results very similar to contrast-enhanced breast MRI [4, 5]. However, no prospective study has yet demonstrated the ability of DWI to screen for breast cancer in women with dense breasts. One reason is the lack of technical robustness of the DWI technique, mainly presenting as artifacts due to insufficient fat suppression and image noise [6]. Given basic MRI physics, it seems evident that these issues should be more aggravated in women with fatty breasts, and less severe in MRI screening aiming at women with dense breasts. For the sake of readability, we refer to the amount of fibroglandular tissue as “breast density,” though this is not entirely the same because MRI does not measure density.

To test the hypothesis that image quality of DWI is higher in women with a high amount of fibroglandular breast tissue, we designed a retrospective bicentric study with multiple readers and compared the image quality of DWI and contrast-enhanced breast MRI stratified by the amount of fibroglandular tissue as a measure of breast density.

## Methods

### Study design

This bicentric visual grading analysis study was performed as a retrospective, cross-sectional, multi-reader diagnostic image quality assessment study at site 1 (University hospital Groningen, the Netherlands) and site 2 (Allgemeines Krankenhaus, Medical University of Vienna, Austria). Both local medical ethical committees waived the need for informed consent due to the retrospective nature of the study. At both sites, a consecutive sample of 200 breast MRIs of female subjects without a history of breast surgery or breast prosthesis was retrospectively included, adding up to 400 included scans. Mean age of included women was 46 years (±13, range 19–83), 42 years at site 1 and 50 years at site 2. Scans of each site were read by two local readers (5th year residents specializing in breast radiology). All readers individually scored 125 scans. Reader 1 read scans 1–125 (site 1), reader 2 read scans 76–200 (site 1), reader 3 read scans 201–325 (site 2), and reader 4 read scans 276–400 (site 2). Thereby, on both sites, 50 cases were double read to check for inter-observer agreement. All readers were blinded to the study aim, hypothesis, original radiology report, and pathology. Indications for scanning were mostly screening in high-risk women. Scans were performed in 2016–2019 for site 1 and 2019–2020 for site 2.

### Breast MRI protocol

Both sites used 2 scanner types, randomly assigned by scanner availability at the hospital. Both 1.5-T (Both sites: Avanto_fit) and 3.0-T (Site 1: Skyra, Site 2: Prisma) MRI scanners (Siemens Healthineers) were used. Reading protocol consisted of T2 with fat suppression, DCE-T1 early post-contrast images (1st series after contrast administration at around 70 s), and diffusion-weighted images.

Site 1:

o DCE-T1-SPAIR: slice thickness 1.2 mm, FOV 350 mm, TR 5.27 ms (1.5 T)/4.50 ms (3.0 T), TE 2.39 ms (1.5 T)/1.63 ms (3.0 T). DCE-T1-DIXON: slice thickness 1.2 mm, FOV 380 mm, TR 5.41 ms, TE 2.46 and 3.69 ms.
o DWI-SS-EPI-SPAIR: slice thickness 4 mm, FOV 350 mm, TR 5500 ms (1.5 T)/6900 ms (3.0 T), TE 64 ms (1.5 T)/65 ms (3.0 T). *b*-values 0, 50, 200, 500, 800, 1000 s/mm^2^. For the ADC map, *b*-values 0 and 1000 s/mm^2^ were used.

Site 2:

o DCE-T1 DIXON: slice thickness 2mm, FOV 360 mm (1.5 T)/340 mm (3.0 T), TR 10 (1.5 T)/3.91 ms (3.0 T), TE 2.39 and 4.77 ms (1.5 T)/1.29 and 2.52 ms (3.0 T).
o DWI-SPAIR: slice thickness 3.5mm (1.5 T)/3 mm (3.0 T), FOV 408 mm (1.5 T)/360 mm (3.0 T), TR 5900 (1.5 T)/4000 (3.0 T), TE 105 (1.5 T)/91 ms (3.0 T). *b*-values 0, 20, 30, 50, 100, 200, 400, 800, and 1500 s/mm^2^ (3.0 T) and 0 and 800 s/mm^2^ (1.5 T). For the ADC map, *b*-values 0 and 800 s/mm^2^ were used.

At site 1, DCE-T1 SPAIR series were randomly replaced by DCE-T1 DIXON series at 2019 in clinic. Therefore, of the included scans, 24 DCE-T1 scans were performed with DIXON fat suppression (six cases were ACR breast density A, 1 case density B, 14 cases density C, and 3 cases density D).

### Visual grading image analysis

Visual grading analysis was performed using ordinal scales [7]. Readers started with scoring the ACR BI-RADS breast density as follows: A = almost entirely fatty, B = scattered areas of fibroglandular tissue, C = heterogeneously dense, D = extremely dense. For the sake of readability, we refer to the amount of fibroglandular tissue as “breast density” throughout the paper though this is not entirely the same and MRI does not measure density. In order to account for confounders, age of the women, background parenchymal enhancement (BPE), and the presence or absence of enhancing lesions (including small spots/foci besides BPE) or cysts were assessed. Enhancing lesions on the DCE-T1 and cysts on the T2 images were scored as one out of 4 options: none/only a few/moderate amount/a lot. BPE was scored based on the ACR BI-RADS lexicon as follows: minimal/mild/moderate/marked. Overall image quality and fat suppression quality of both DCE-T1 and ADC/DWI images were scored on a standard 5-point Likert scale of 1 to 5, in which 1 = a minimal/non-diagnostic quality and 5 = maximal quality. General image quality was based on visual assessment of signal, noise, contrast, sharpness, artifacts (ghosting), and the presence of anatomical distortions.

### Statistical analysis

To test for inter-observer agreement, Kappa statistics was performed to check for agreement between readers 1 and 2 and between readers 3 and 4, who double read 50 cases of their research site. Kappa 0 = agreement fully based on chance; kappa 1 = complete inter-observer agreement. Thereafter, data of all 4 readers were combined. Cross tabulations were generated to visualize the differences in image and fat suppression quality of the different categories of fibroglandular tissue composition. Visual grading characteristic (VGC) curves and their area under the VGC curve (AUC) were calculated for fatty breasts (A/B) versus dense breasts (C/D) based on the theory of Bath et al [7]. Area under the VGC curve was also calculated for 1.5 vs. 3.0 T. IBM SPSS Statistics 24 was used for statistical analysis.

## Results

### Population characteristics

Of the 400 scans, 71 (18%) were scored as breast density A, 125 (31%) as B, 112 (28%) as C, and 92 (23%) as D. At both sites, 100 scans were performed at 1.5 T and 100 scans at 3.0 T. Table 1 shows the homogeneous breast density distribution of the two sites, separately visualized for 1.5- and 3.0-Tesla scanners.

**Table 1:** Distribution of the amount of fibroglandular tissue on 1.5 and 3.0 T for sites 1 and 2 separately. *T* Tesla

	Site 1	Site 2	Total
1.5 T			
Almost entirely fat	21 (%)	9 (%)	30 (15%)
Scattered fibroglandular tissue	31 (%)	35 (%)	66 (33%)
Heterogeneous fibroglandular tissue	22 (%)	29 (%)	51 (25.5%)
Extreme fibroglandular tissue	26 (%)	27 (%)	53 (26.5%)
Total	100	100	200
3.0 T			
Almost entirely fat	18 (%)	23 (%)	41 (20.5%)
Scattered fibroglandular tissue	28 (%)	31 (%)	59 (29.5%)
Heterogeneous fibroglandular tissue	33 (%)	28 (%)	61 (30.5%)
Extreme fibroglandular tissue	21 (%)	18 (%)	39 (19.5%)
Total	100	100	200

### Inter-observer agreement

There was a good inter-observer agreement of breast density for readers 1 and 2 (site 1) (kappa = 0.595) and for readers 3 and 4 (site 2) (kappa = 0.734). Kappa values were 0.427 and 0.199 for image quality of T1 early post-contrast and 0.423 and 0.480 for quality of fat suppression of DCE-T1, respectively. Kappa values for image quality of DWI were −0.012 (± 0.109) and 0.498 (±0.090) and for fat suppression quality of DWI −0.033 (± 0.059) and 0.232 (±0.085). The negative values can be explained by differences in preferences of observers for using the extreme values on the Likert scale (1 or 5).

### Breast density and image quality

In DWI, image quality was higher with ascending breast density, with good scores (score 3–5) for 85.9% of density D and 88.4% of density C cases, compared to 61.6% for density B and 53.5% for density A cases. High scores (score 4) for image quality of DWI were more common in breast density C and D compared to A and B (Table 2). In the comparison between dense breasts (C/D) versus fatty breasts (A/B), the VGC curve for overall image quality of DWI was in favor of dense breasts (C/D), with an area under the VGC curve of 0.659 (*p* < 0.001).

**Table 2:** Overall image quality for each amount of fibroglandular tissue category in absolute values (and percentages). *DWI* diffusion-weighted imaging, *DCE* dynamic contrast enhanced

	Almost entirely fat	Scattered fibroglandular tissue	Heterogeneous fibroglandular tissue	Extreme fibroglandular tissue	Total
Overall image quality of DWI images					
1 (minimal/non-diagnostic)	15 (21.1%)	16 (12.8%)	4 (3.6%)	5 (5.4%)	40
2	18 (25.4%)	32 (25.6%)	9 (8%)	8 (8.7%)	67
3	15 (21.1%)	39 (31.2%)	49 (43.8%)	26 (28.3%)	129
4	23 (32.4%)	37 (29.6%)	46 (41.1%)	52 (56.5%)	158
5 (maximal)	0 (0%)	1 (0.8%)	4 (3.6%)	1 (1.1%)	6
Total:	71 (100%)	125 (100%)	112 (100%)	92 (100%)	400
Overall image quality of T1-DCE					
1 (minimal/non-diagnostic)	1 (1.4%)	1 (0.8%)	0 (0%)	0 (0%)	2
2	1 (1.4%)	3 (2.4%)	0 (0%)	1 (1.1%)	5
3	8 (11.3%)	12 (9.6%)	10 (8.9%)	7 (7.6%)	37
4	45 (63.4%)	79 (63.2%)	65 (58%)	47 (51.1%)	236
5 (maximal)	16 (22.5%)	30 (24%)	37 (33%)	37 (40.2%)	120
Total:	71 (100%)	125 (100%)	112 (100%)	92 (100%)	400

Area under the VGC curve for overall image quality T1 early post-contrast was 0.574 (*p* = 0.011).

Overall image quality of DCE-T1 was good, with comparable percentages of maximal scores (score 5) for breast density C and D (33% and 40.3%, respectively) compared to breast density A and B (22.5 and 24%, respectively) (Table 2). Image quality score 4 for T1-DCE was also comparable for breast density A, B, C, and D, with values of 63.4%; 63.2%; 58.0%; and 51.1% respectively.

### Breast density and fat suppression quality

**Fig. 1:**
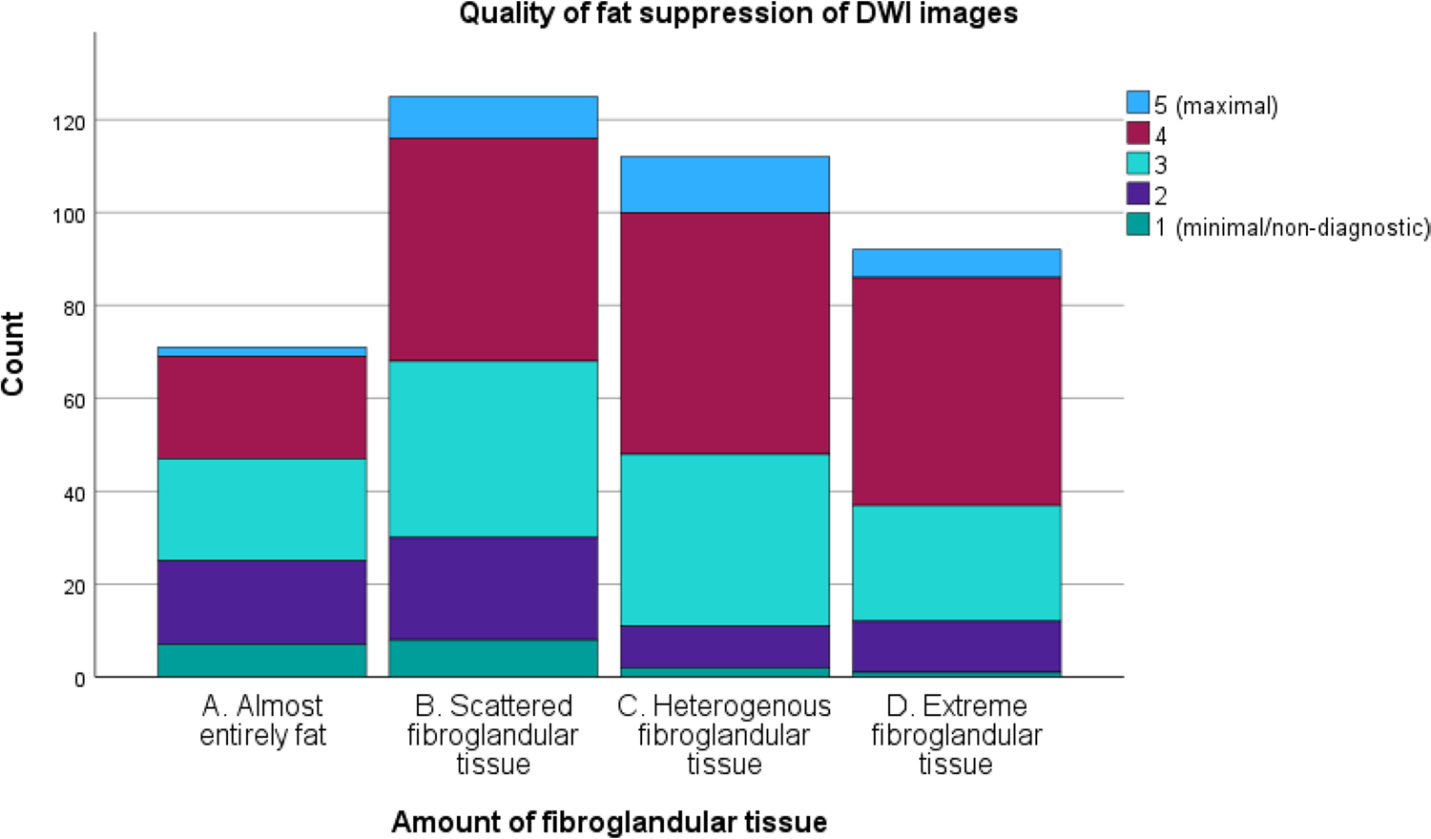
Stacked bar chart of the quality of fat suppression of diffusion-weighted imaging for each amount of fibroglandular tissue category

**Fig. 2:**
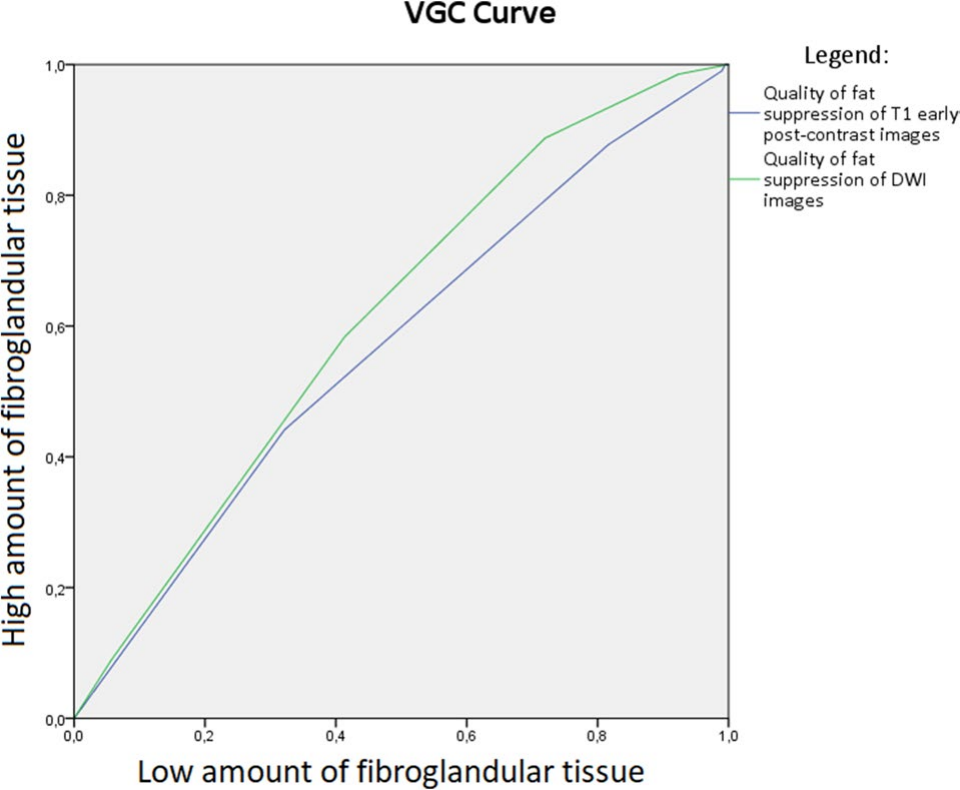
Visual grading characteristics (VGC) curve comparing the fat suppression quality of DWI images and T1 early post-contrast images between breast with a low and a high amount of fibroglandular tissue

In DWI, quality of fat suppression increased with higher breast density (Fig. 1) with good scores (score 3–5) for 86.9% of density D and 90.2% of density C cases compared to 76.0% for density B and 54.7% for density A cases. A high score of 4 was given to 53.3% of density D, 46.4% of density C, 38.4% of density B, and only 31% of density A breasts. The VGC curve visualizes this (relatively small difference), with an AUC of 0.615 (*p* < 0.001) for DWI images, favoring dense breasts (category C/D) (Fig. 2).

**Fig. 3:**
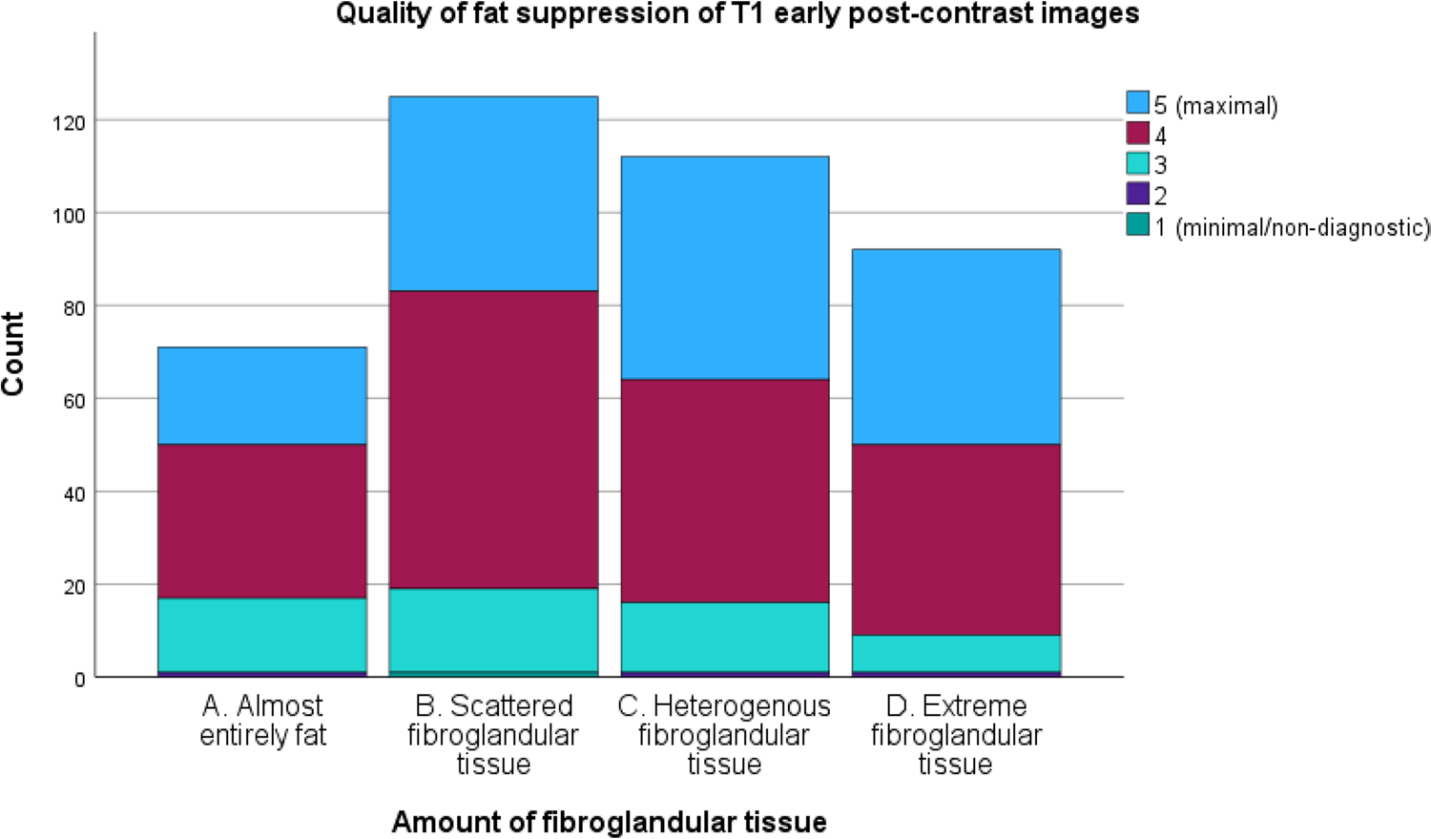
Stacked bar chart of the quality of fat suppression of DCE-T1 for each amount of fibroglandular tissue category

Quality of fat suppression of T1-DCE was also higher for breast density C/D compared to that for A/B, with, for example, higher percentages for maximal quality (score 5) of 45.7% for density D, 42.9% for density C, 33.6% for density B, and 29.6% for density A. Figure 3 shows this result in a stacked bar chart. The VGC curve showed an AUC of 0.569 (*p* = 0.016) or fat suppression quality of T1-DCE, favoring dense breasts (Fig. 2).

### Comparison of fat suppression types in DCE-T1

**Fig. 4:**
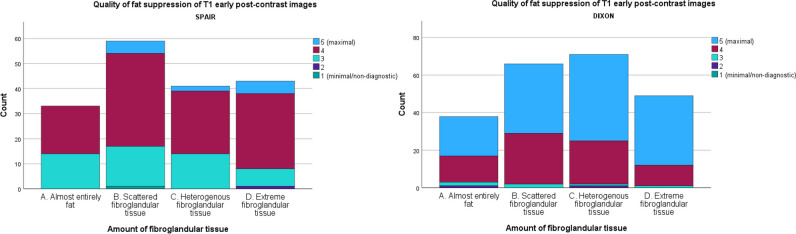
Stacked bar chart showing the quality of fat suppression of DCE-T1 for DIXON and SPAIR fat suppression, displayed per amount of fibroglandular tissue category

In total, 224 DCE-T1 scans were performed with DIXON fat suppression (all scans of site 2 and 24 of site 1) and 176 with SPAIR (site 1). Fat suppression quality was higher for DIXON, with 52.7% of total scans scoring maximal, versus 1.1% for SPAIR. Higher fat suppression quality for DIXON compared to SPAIR was seen in all breast density categories (Fig. 4).

### Comparison of field strengths

In the comparison of image quality between 1.5 T and 3.0 T, there was a slight bend of the VCG curve towards 3.0 T, with an AUC of 0.561 (*p* = 0.029) for DCE-T1 and 0.570 (*p* = 0.015) for DWI. There was no difference for fat suppression quality of DWI (AUC 0.553, *p* = 0.066) between 1.5 and 3.0 T. Fat suppression quality of DCE-T1 was in favor of 3.0 T (AUC 0.617, *p* < 0.001).

## Discussion

Our study demonstrates that image quality of DWI depends on breast density, specifically on the amount of fibroglandular tissue (FGT). Dense breasts (ACR C/D) had higher scores on both overall image quality and fat suppression quality of DWI, as pointed out in the VGC curves bending towards dense breasts (Fig. 2). These results are important as they explain the heterogeneous results of DWI in terms of diagnostic image quality in literature, which led to the assumption that DWI in general is not robust. In fact, the majority of DWI scans performed in two centers and with four MRI scanners (both 1.5 T and 3.0 T) were of diagnostic quality. This emphasizes the fact that DWI indeed may be tested for screening purposes already. Dense breasts also showed high overall DWI image quality scores compared to fatty breast. On a side note, contrast-enhanced T1-weigthed images did not show a relevant dependence on the amount of FGT.

In regard to the recent developments of tailored breast cancer screening and the advice to apply supplemental screening with MRI in extremely dense breasts, it is important to find a fast and convenient scan protocol [1, 8, 9]. It has been shown that MRI as solitary screening technique in extremely dense breast is cost-effective, especially when applying a 4-year interval. However, some authors argue in favor of shorter, still cost-effective, screening intervals [10–12]. Nonetheless, the need for contrast injection constitutes an invasive screening approach. Therefore, a DWI screening protocol that avoids IV contrast would be more feasible and less invasive. The high image and fat suppression quality of DWI in women with dense breasts (which would be the population to screen) is encouraging.

Until now, only the influence of breast density on conventional imaging such as mammography (MG) is studied and awareness of the negative impact of higher breast density on the detection rate is growing [13]. However, in breast MRI, only the independence of contrast-enhanced imaging diagnostic outcomes from breast density has been demonstrated [14]. There is no comparable literature on the influence of breast density on DWI quality or diagnostic performance. Regarding DWI image quality, only data addressing the influence of different *b*-values, synthetic *b*-values, or different DWI and fat suppression techniques have been studied previously [15–19].

General image quality, as rated by the 4 readers, is based on signal, noise, contrast, sharpness, artifacts (ghosting), and the presence of anatomical distortions. While in T1-DCE fat suppression may help image interpretation, it is mandatory for valid image interpretation in DWI. This extra step in image acquisition gives rise to more artifacts, distortions, or areas of incomplete fat suppression. These issues depend on the amount of fatty tissue to be suppressed and thus explain our results showing higher image and fat suppression quality in dense breasts.

In T1-DCE imaging, a subgroup analysis compared fat suppression quality between SPAIR and DIXON, showing higher fat suppression quality for DIXON with 52.7% maximal scores (score 5) compared to 1.1% for SPAIR. We therefore recommend DIXON fat suppression for T1-DCE in clinical breast MRI, leading to better image quality independent of B0 inhomogeneity without the need for extra scanning time. This is in concordance with the results of Kalovidouri et al, who studied both the imaging quality and signal to noise ratio and confirmed the superiority of DIXON (at 3.0 T) compared to SPAIR in breast imaging [20]. Clauser et al also concluded that the DIXON technique outperformed a spectral water suppression technique in all evaluated visual grading criteria in breast T1-DCE at 3.0 T [18], in analogy to musculoskeletal tumor imaging [21].

Furthermore, this study showed no influence of the field strength on the image or fat suppression quality. In literature, it is suggested that fat suppression is more difficult at 3.0 T compared to that at 1.5 T [22]. Breast size, which may influence fat suppression, was not available and therefore not taken into consideration.

One of the qualities of the study is the high sample size and the multicentric multi-reader approach. A limitation of a visual grading analysis study is the impossibility to know the exact image characteristics or image disturbances on which the grading score was based for each reader. However, the good inter-observer agreement of DWI imaging showed the consistency of the scoring and its validity. Another limitation is the non-diagnostic approach, with image quality as a surrogate. We did for instance not investigate the dependence of fibroglandular tissue on lesion conspicuity as the visibility of breast lesions using unenhanced DWI-based imaging has been since long established and as there are no physical reasons why FGT should or even could have such an influence. However, these results are sufficient to refute the notion of “DWI is not robust,” by revealing a major influencing factor on DWI quality.

With the help of automated breast density scoring and possible other deep learning applications for excluding normal scans, DWI could be further tested as an additional screening method in dense breasts [23, 24]. Future research is needed to optimize fat suppression protocols for non-dense breasts and develop deep learning algorithms for breast cancer screening with DWI.

## Conclusion

Dense breasts show excellent fat suppression and substantially higher image quality in DWI images compared with non-dense breasts. These results support the setup of studies exploring DWI-based MR imaging without IV contrast for additional screening of women with dense breasts.

### Supplementary Information

Below is the link to the electronic supplementary material.

Supplementary file1 (PDF 223 KB)

## Abbreviations

ADC: Apparent diffusion coefficient
AUC: Area under the curve
BPE: Background parenchymal enhancement
DCE: Dynamic contrast enhanced
DWI: Diffusion-weighted imaging
FGT: Fibroglandular tissue
FOV: Field of view
IV: Intravenous
VCG: Visual grading characteristic

## Declarations

### Guarantor

The scientific guarantor of this publication is Assoc. Prof. Priv.-Doz. Dr. Pascal A. T. Baltzer.

### Statistics and biometry

One of the authors has significant statistical expertise.

### Study subjects or cohorts overlap

No study subjects or cohorts have been previously reported.

### Methodology

• retrospective

• cross-sectional study

• performed at two institutions

## References

[1] Bakker MF, de Lange SV, Pijnappel RM (2019). Supplemental MRI screening for women with extremely dense breast tissue. N Engl J Med.

[2] Mann RM, Athanasiou A, Baltzer PAT, et al (2022) Breast cancer screening in women with extremely dense breasts recommendations of the European Society of Breast Imaging (EUSOBI). Eur Radiol 4036–4045. 10.1007/s00330-022-08617-6 10.1007/s00330-022-08617-6 PMC9122856 35258677

[3] McCormack VA, Dos Santos Silva I (2006). Breast density and parenchymal patterns as markers of breast cancer risk: a meta-analysis. Cancer Epidemiol Biomarkers Prev.

[4] Bickelhaupt S, Laun FB, Tesdorff J (2016). Fast and noninvasive characterization of suspicious lesions detected at breast cancer X-ray screening: capability of diffusion-weighted MR imaging with MIPs. Radiology.

[5] Kang JW, Shin HJ, Shin KC (2017). Unenhanced magnetic resonance screening using fused diffusion-weighted imaging and maximum-intensity projection in patients with a personal history of breast cancer: role of fused DWI for postoperative screening. Breast Cancer Res Treat.

[6] Le Bihan D, Poupon C, Amadon A, Lethimonnier F (2006). Artifacts and pitfalls in diffusion MRI. J Magn Reson Imaging.

[7] Ba M, Båth M, Månsson LG (2007). Visual grading characteristics (VGC) analysis: a non-parametric rank-invariant statistical method for image quality evaluation. Br J Radiol.

[8] Gilbert FJ, Hickman SE, Baxter GC (2021). Opportunities in cancer imaging: risk-adapted breast imaging in screening. Clin Radiol.

[9] Wengert GJ, Helbich TH, Kapetas P (2018). Density and tailored breast cancer screening: practice and prediction – an overview. Acta Radiol Open.

[10] Froelich MF, Kaiser CG (2021). Cost-effectiveness of MR-mammography as a solitary imaging technique in women with dense breasts : an economic evaluation of the prospective TK-Study. Eur Radiol.

[11] Geuzinge HA, Bakker MF, Heijnsdijk EAM (2021). Cost-effectiveness of magnetic resonance imaging screening for women with extremely dense breast tissue. J Natl Cancer Inst.

[12] Tollens F, Baltzer PAT, Froelich MF, Kaiser CG (2022). Comment on: Cost-effectiveness of magnetic resonance imaging screening for women with extremely DENSE breast tissue. Eur J Radiol.

[13] Rhodes DJ, Breitkopf CR, Ziegenfuss JY (2015). Awareness of breast density and its impact on breast cancer detection and risk. J Clin Oncol.

[14] Berg WA, Gutierrez L, NessAiver MS (2004). Diagnostic accuracy of mammography, clinical examination, US, and MR imaging in preoperative assessment of breast cancer. Radiology.

[15] Choi BH, Baek HJ, Ha JY (2020). Feasibility study of synthetic diffusion-weighted MRI in patients with breast cancer in comparison with conventional diffusion-weighted MRI. Korean J Radiol.

[16] Han X, Li J, Wang X (2017). Comparison and optimization of 3.0 T breast images quality of diffusion-weighted imaging with multiple b-values. Acad Radiol.

[17] Baltzer PAT, Renz DM, Herrmann K-H (2009). Diffusion-weighted imaging (DWI) in MR mammography (MRM): clinical comparison of echo planar imaging (EPI) and half-Fourier single-shot turbo spin echo (HASTE) diffusion techniques. Eur Radiol.

[18] Clauser P, Pinker K, Helbich TH (2014). Fat saturation in dynamic breast MRI at 3 Tesla: is the Dixon technique superior to spectral fat saturation? A visual grading characteristics study. Eur Radiol.

[19] Dorrius MD, Dijkstra H, Oudkerk M (2014). Effect of b value and pre-admission of contrast on diagnostic accuracy of 1.5-T breast DWI: a systematic review and meta-analysis. Eur Radiol.

[20] Kalovidouri A, Firmenich N, Delattre BMA (2017). Fat suppression techniques for breast MRI: Dixon versus spectral fat saturation for 3D T1-weighted at 3 T. Radiol Med.

[21] Huijgen WHF, van Rijswijk CSP, Bloem JL (2019). Is fat suppression in T1 and T2 FSE with mDixon superior to the frequency selection-based SPAIR technique in musculoskeletal tumor imaging?. Skeletal Radiol.

[22] Lin C, Rogers C, Majidi S (2015). Fat suppression techniques in breast magnetic resonance imaging: a critical comparison and state of the art. Reports Med Imaging.

[23] van der Velden BHM, Janse MHA, Ragusi MAA (2020). Volumetric breast density estimation on MRI using explainable deep learning regression. Sci Rep.

[24] Codari M, Schiaffino S, Sardanelli F, Trimboli RM (2019). Artificial intelligence for breast MRI in 2008–2018: a systematic mapping review. AJR Am J Roentgenol.

