# Supplementary material for: Image quality of DWI at breast MRI depends on the amount of fibroglandular tissue: implications for unenhanced screening
Source: Eur Radiol. 2023 Nov 27;34(7):4730–7. doi: 10.1007/s00330-023-10321-y (PMC11213722; doi:10.1007/s00330-023-10321-y)

Supplemental Figure 1. Violin plot showing the quality of fat suppression of DWI for each category of cysts (0= none, 1= only a few, 2= moderate amount, 3= a lot).

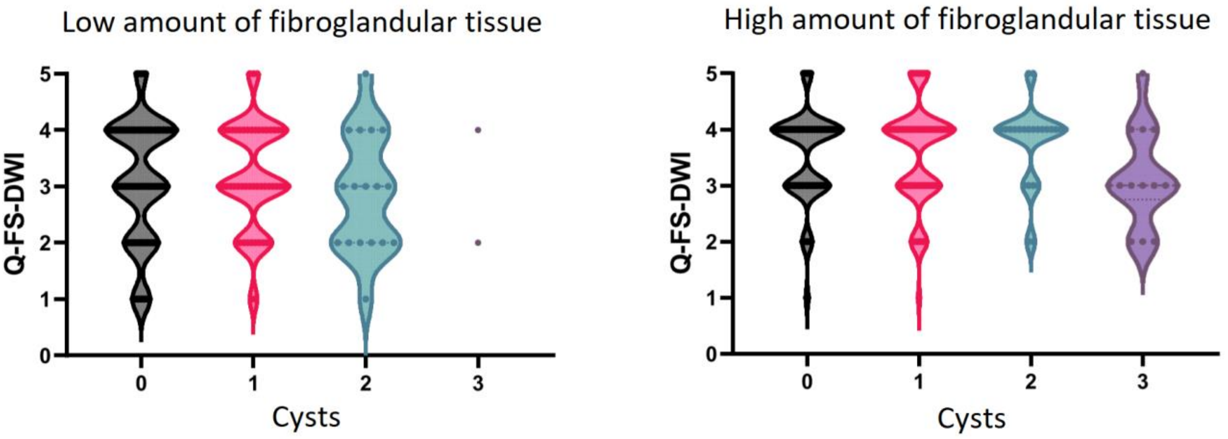

Supplemental Figure 2. Violin plot showing the quality of fat suppression of DWI for each category of background parenchymal enhancement (BPE) (0= minimal, 1= mild, 2= moderate, 3= marked).

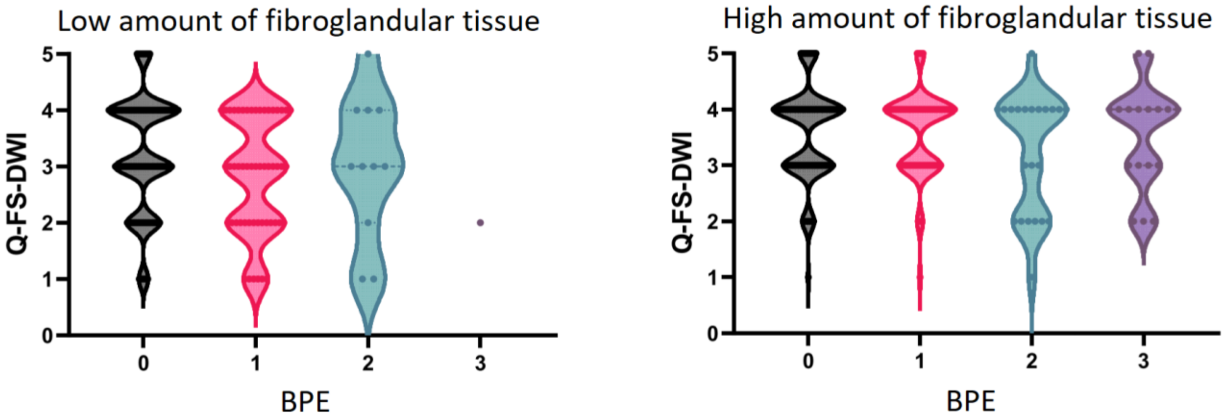

Supplement: Supplementary file 1 — Supplementary file1 (PDF 223 KB) [file 330_2023_10321_MOESM1_ESM.pdf]
